# Potential factors result in diminished ovarian reserve: a comprehensive review

**DOI:** 10.1186/s13048-023-01296-x

**Published:** 2023-10-25

**Authors:** Qinying Zhu, Yi Li, Jianhong Ma, Hao Ma, Xiaolei Liang

**Affiliations:** 1 The First Clinical Medical College of Lanzhou University, Lanzhou, China; 2 Department of Obstetrics and Gynecology, Key Laboratory for Gynecologic Oncology Gansu Province, The First Hospital of Lanzhou University, No.1, Donggangxi Rd, Chengguan District, 730000 Lanzhou, China

**Keywords:** Diminished ovarian reserve, Hormone, Metabolism, Primordial follicular pool, Environmental factors

## Abstract

The ovarian reserve is defined as the quantity of oocytes stored in the ovary or the number of oocytes that can be recruited. Ovarian reserve can be affected by many factors, including hormones, metabolites, initial ovarian reserve, environmental problems, diseases, and medications, among others. With the trend of postponing of pregnancy in modern society, diminished ovarian reserve (DOR) has become one of the most common challenges in current clinical reproductive medicine. Attributed to its unclear mechanism and complex clinical features, it is difficult for physicians to administer targeted treatment. This review focuses on the factors associated with ovarian reserve and discusses the potential influences and pathogenic factors that may explain the possible mechanisms of DOR, which can be improved or built upon by subsequent researchers to verify, replicate, and establish further study findings, as well as for scientists to find new treatments.

## Introduction

Ovarian reserve is the quantity of primordial follicles and follicles that can be recruited into the pre-antral and antral stages and are competent for ovulation [1]. Women have inherent and finite number of ovarian follicles that are gradually descend during their reproductive years until poor reproductive outcome occurs [2]. The growth of ovarian follicles composes the basis of female reproduction, and the proliferation of granulosa cells (GCs) is a foundational process required to ensure normal follicular development [3]. Clinicians consider several ovarian reserve tests, including biochemical tests, such as follicle-stimulating hormone (FSH), luteinizing hormone (LH), estradiol (E_2_), inhibin B, and antimüllerian hormone (AMH), and ultrasound imaging of the ovaries like antral follicle count (AFC) [4, 5]. As a complex clinical sign, ovarian reserve can be influenced by many factors such as age, environment, primordial follicular pool, diseases and drugs, and some unknown elements [1, 6]. Compared with the normal group at a similar age, the number of available follicles or oocytes decreased to a very poor count in women with DOR [5]. With the progress of modern society and the postponement of pregnancy, DOR has become an intractable problem for women eager to become pregnant and also for the societal environment of the next generation [7]. Thus far, the etiology and pathogenesis of DOR are still vague; therefore, this review details about the variable factors that could affect ovarian reserve and hopes to improve our understanding of DOR by reorganizing its effects, putting forward some potential reasons for DOR, and hoping to have some impact on future clinical treatment.

## Hormonal influence on ovarian reserve

### AMH

As one of the most commonly used clinical markers to estimate ovarian reserve, AMH level is a reliable index that reflects the pool of follicles in the gonadotropin-independent phase and ovarian follicle count [5, 8, 9]. Accumulated AMH plays a key role in facilitating follicular growth from the pre-antral to the antral phase [10]. In antral follicles, AMH secretion gradually decreases from a peak to undetectable levels at 8–10 mm follicle diameter [11]. Furthermore, AMH levels appeared to reflect the number of growing and primordial follicles [12]. Thus, AMH plays an important role in the recruitment of primordial follicles, and the absence of the AMH gene results in more primordial follicles in young mice and fewer in older mice [13], suggesting that AMH deficiency depletes the resting pool, causing successively low AFC in the early years and finally inducing DOR. The local effects of AMH on folliculogenesis may also be stage-dependent. Additionally, the gene of AMH expression in GCs is positively associated with AMH levels and the expression of the FSH receptor (FSHR), androgen receptor (AR) and AMH receptor (AMHR), and negatively associated with the levels of estradiol (E_2_) and progesterone (P) in the corresponding follicular fluid, suggesting a reflective function of AMH concentration in synchronous follicular growth [14]. The same study also indicated that AMH levels in the circulation reflect the constitution of follicles ranging from 5 to 8 mm in diameter.

### FSH and LH

The quantity of small antral follicles at the initiation of each follicle development cycle is a manifestation of the ovarian reserve. Sufficient FSH production is required for the growth of smaller follicles, which is related to follicle recruitment [15, 16]. The presence of FSH is crucial after the pre-antral follicle stage, although the correct level of FSH at the pre-antral stage is associated with greater oocyte quality [17]. Sustained and sufficient stimulation of FSH is key to the action of aromatase enzymes and estradiol-dependent follicular development; therefore, it is initially at the stage of large antral follicles at the onset of ovulation [18]. In vitro experiments have shown an association between FSH and steroid production, substance exchange, and metabolism, which ultimately induces follicle maturation [19].

Adequate LH secretion is necessary for the further maturation of follicles, especially preovulatory follicles [18, 20]. Research has shown that appropriate stimulation of LH is required for follicular growth by regulating the action of steroids on ovarian cells; however, excess LH could lead to the inhibition of the differentiation and proliferation of GCs [18]. Furthermore, the initial number of antral follicles chosen for ovulation is highly dependent on the distribution density and regulatory activity of the FSHR and LHR on the membrane of GCs [16, 21]. Moreover, adequate FSHR and LHR are required for further follicle maturation by activating the conversion of androstenedione to estrogen [15]. The quality of oocytes can be compromised by a low density of FSH and insufficient expression of FSHR and LHR [22].

### Inhibin B

Inhibin B is secreted by growing follicles, accumulates in the follicular compartment, and is released into circulation. Its primary function is to selectively inhibit the synthesis and secretion of FSH and activate LH. As it is a GC-specific hormone, its intrafollicular concentration is related to the size of the follicle, and its serum level could reflect follicle synthesis. Inhibin B is more effective in predicting women with AFC ranging from 5 to 7 mm [23]. Inhibin B summits during the follicle growth reaching a diameter of 8–10 mm [11], and the episodic inhibin B secretion pattern shows a peak during the follicular phase and ovulation [24]. Andersen hypothesized that inhibin B participates in the selection of dominant follicles during the natural human menstrual cycle [25]. Inhibin B is secreted in response to follicle-stimulating by FSH and promotes androgen production. Androgens then accumulate in adjacent GCs and upregulate the expression of FSHR and LHR, ultimately leading to increased sensitivity to gonadotropins in the follicle and further follicular growth.

### Estrogen, progesterone and androgens

There is E_2_-dependent, calcium-mediated cytoplasmic maturation in human oocytes [26]. A study found that follicular fluid (FF) obtained from dominant follicles had a higher estrogen/androgen ratio than atretic subordinate follicles, indicating that well-developed follicles require abundant estrogen [16]. Estrogen receptor (ER) plays an important role in follicle development beyond the antral stage by regulating the transcriptional levels of genes involved in the communication between GCs and oocyte, oocyte maturation and gonadotropin-induced follicle development [20, 27].

Additionally, progesterone secretion gradually increases during antral follicle development [11], surges in the preovulatory period, and peaks in the dominant follicles [28]. Abnormally high levels of both P and E_2_ can negatively regulate follicle development from the primordial to the primary stage and impair mitosis of GCs, suggesting the arrest of primordial follicle growth [29, 30]. Thus, the over secretion of P and E_2_ at the early stage of follicular development affects the ovarian reserve.

Androgens exert complex effects on follicular development. The expression of AR in GCs is enriched in the pre-antral and early antral stages of follicles and is progressively absent in late preovulatory follicles [31, 32]. The absence of AR results in the apoptosis of GCs and subsequently compromises the ovarian reserve [33, 34].

### Spatiotemporal pattern of hormone-mediated folliculogenesis

**Fig. 1:**
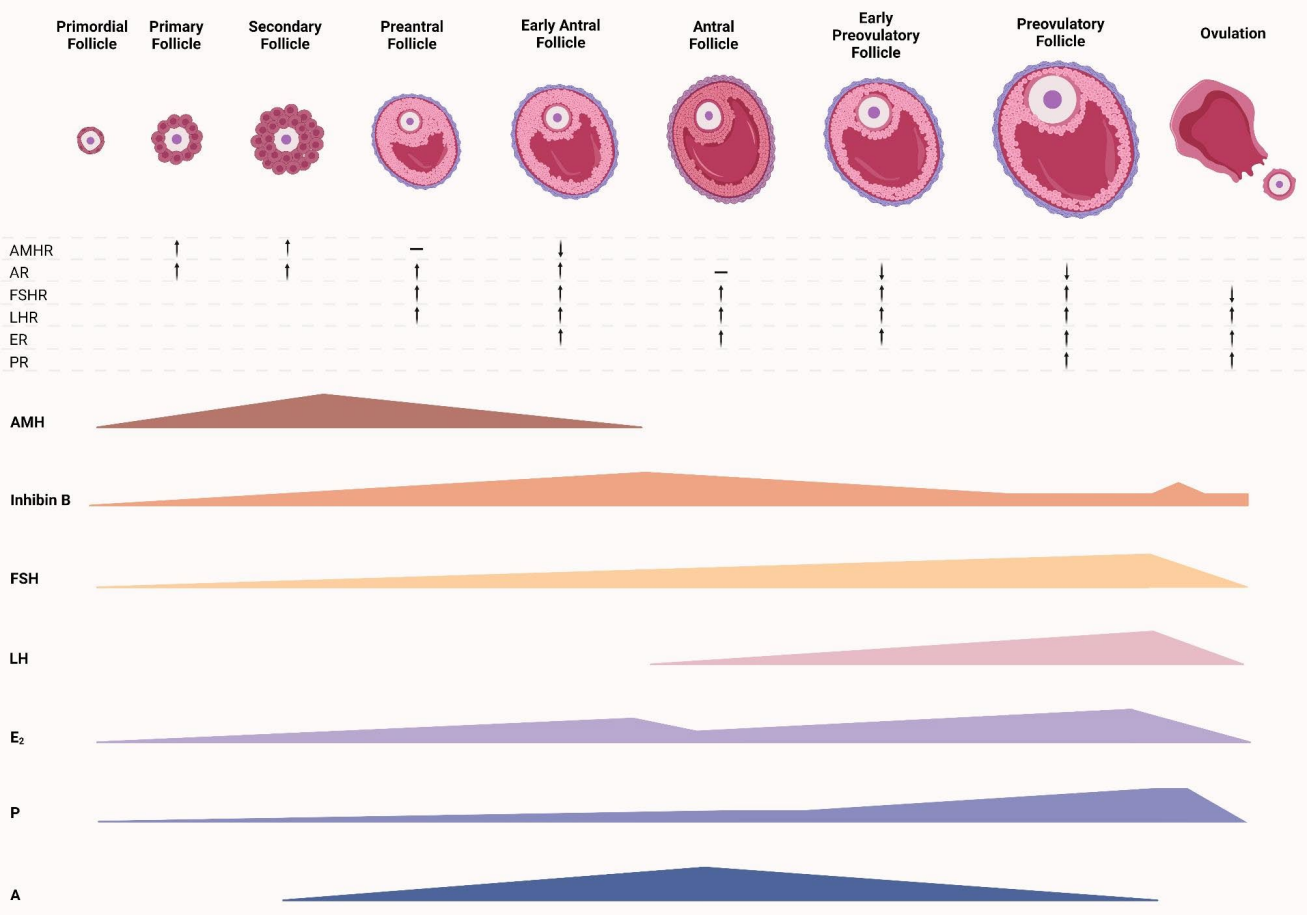
Model of well-orchestrated spatiotemporal pattern of hormone-mediated folliculogenesis. AMH expressed in the stage from primordial to early antral, and decreased in antral stage. AMH plays an important role in the recruitment of primordial follicles. The decline of AMH can enhance the secretion of FSH, LH, and A. The expression of AMH can be finally inhibited by E2 and P. FSH mainly appears after pre-antral follicle stage caused by the decline of AMH and peaks before ovulation, and its sufficient secretion increased E2 level. LH secretion is induced by the decline of inhibin B at antral follicle stage and peaks before ovulation. LH can enhance the secretion of E2. Inhibin B increases before antral follicle stage, drops to a low level after the same stage, and surges again during ovulation. Its secretion is stimulated by FSH and up-regulates androgens. Progesterone secretion gradually increases during antral follicle development, surges in the preovulatory period, and peaks in the dominant follicles. The variation of different receptor is consistent in the expression of corresponding hormones

From the perspective of synchronized follicular development, a well-orchestrated spatiotemporal pattern of hormone-mediated folliculogenesis is crucial for ovarian reserve. One of the basic functions of AMH in folliculogenesis is the transition of follicles from the FSH-independent to the FSH-dependent stage, that is, the period from pre-antral to antral follicle stages [10]. FSH-stimulated pre-antral follicle development can be inhibited by AMH, and a lack of FSH induces attenuation of primordial follicle recruitment, suggesting that AMH can regulate the sensitivity of early follicles to FSH [35], and over secretion of AMH can lead to a dramatic drop in the number of follicles after the primary stage [36]. The tight association between AMH and AMHR expression in GCs suggests that cells and receptors are involved in the synchronous mechanisms that may influence follicular development. This is further supported by the significant association between AMHR and FSHR [14]. During dominant follicular selection, the secretion of E_2_ and P increases rapidly, and the concentration of inhibin B reaches its peak [11]. In contrast, the AMH level drops to its lowest point, indicating the cooperation of these hormones [11]. A high concentration of AMH during the early period of follicular growth also suppresses the secretion of E_2_, which is related to preovulatory follicle selection, by eliminating its function by acting on the aromatase of FSH [10]. Moreover, an appropriate androgen concentration is beneficial for FSH-dependent pre-antral follicular development by augmenting the expression of FSH at the mRNA level [37]. Therefore, as shown in Fig. 1, an appropriate concentration, opportune action, and synergistic response mechanism of intrafollicular hormones are essential for regulating the development of follicles in relation to the phenotypic ovarian reserve.

### Important metabolic part about ovarian reserve

#### Glucose metabolism

Glucose metabolism is necessary to generate sufficient material for follicle expansion, and an increased glucose requirement is necessary for the proliferation and differentiation of CCs and oocyte development. Because of the hydrophobicity of glucose, GCs absorbed glucose through glucose transporters (GLUTs) [38]. The main pathways for glucose metabolism in cumulus-oocyte complexes (COC) include glycolysis, the pentose phosphate pathway (PPP), the hexosamine biosynthetic pathway (HBP) and the polyol pathway [39]. Glycolysis in human GCs is enhanced from primordial to primary follicle [40]. In the absence of phosphofructokinase, energy production in the oocyte relies on pyruvate provided by GCs, and the oocyte synchronously regulates the high expression of phosphofructokinase in GCs [41]. Prior to follicular maturity, the production of pyruvate and lactate in GCs increases due to high energy consumption [42]. Pyruvate deprivation leads to early follicular dysgenesis [43]. Lactate may play a signaling molecular role in the follicular-luteal transition [44], and a lack of lactate causes follicular dysplasia [45]. There are two main functions of PPP in the COC. The first is the production of NADPH for the operation of the antioxidant system, including the combination of reactive oxygen species (ROS) to reduce cellular oxidation levels [42, 46]. The second function is the generation of phosphoribosyl pyrophosphate (PRPP) to synthesize nucleotides and nucleic acids. In addition, PPP is involved in the meiotic induction mechanism of oocytes [47], and inhibition of PPP can reduce the ratio of MII oocytes, demonstrating that PPP is a key regulator of the nuclear and cytoplasmic maturation of oocytes [48]. Additional glucose enters the HBP to provide a substrate for hyaluronic acid production during extracellular matrix expansion [49, 50]. Finally, a small tiny fraction of the absorbed glucose is used by the GCs to produce sorbitol and fructose via the polyol pathway [51]. Sorbitol, a byproduct of the polyol pathway, can increase superoxide dismutase (SOD) levels, which enhances ROS levels and results in follicular dysmaturity and ovarian aging [52, 53]. However, the presence of sorbitol or fructose in GCs and oocytes has not yet been established.

#### Lipids and cholesterol

Lipids, which are mainly metabolized in CCs around the oocyte, are potential resources for energy production in ovarian cells [54, 55]. Fatty acid β-oxidation, which is induced by the LH surge, plays an initial role in the metabolism of COCs, and the inhibition of fatty acid β-oxidation results in decreased oocyte quality [56]. The concentration of some fatty acids (i.e. pentadecanoic acid, heptadecanoic acid, cis-11-octadecenoic acid, cis-11-eicosenoic acid, cis,cis-11,14-eicosadienoic acid, and behenic acid, ect) were lower in DOR group with statistical significance, and the decreased transcription of HADHA (hydroxyacyl-coenzyme A dehydrogenase), ACSL (fatty acids β-oxidation related genes) were also decreased in DOR patients [57]. Therefore, further studies on the relationship between fatty acids and ovarian reserve are required.

The adiponectin signaling pathway is involved in ovarian activities, such as the regulation of steroid production in GCs and oocyte growth [58]. One possible mechanism of action of adiponectin is the activation of the hypothalamic-pituitary axis, and the absence of adiponectin interferes with the production of FSH and LH and subsequently influences ovulation [59]. Moreover, AdipoRon, an adiponectin-like synthetic, inhibits folliculogenesis and GCs proliferation by regulating aromatase expression, steroid production, and estrogen secretion by increasing the activity of phosphodiesterase, which causes cyclic adenosine monophosphate (cAMP) production [60].

Alterations in steroid hormones are related to oocyte development, and cholesterol participates in steroidogenesis. Studies have shown that intracellular cholesterol concentration is likely to reflect the developmental competence of the oocyte [61, 62]. Furthermore, cholesterol can be converted to progesterone and estradiol, which can further regulate the function of GCs [63, 64], and the synergistic effect of progesterone and estradiol delays oocytes development at an early stage [65]. Genes involved in steroid biosynthesis are different between the DOR and NOR groups [66], and subsequent research demonstrated that cholesterol-related metabolites, including coprostanone, 11 A-acetoxyprogesterone, 17α-hydroxyprogesterone, and estradiol, are decreased in the GCs of the DOR group [67]. Moreover, the transcriptional levels of SCAP, a key gene involved in cholesterol regulation, and CYP19A1, a key gene related to steroidogenesis, were downregulated in the DOR group. Cholesterol synthesis and transport-related genes such as IDI1, FDFT1, CYP51A1, and STARD1, also decreased significantly.

#### Mitochondria

Mitochondrial biogenesis is one of the key intracellular pathways involved in programming the ovarian reserve, and the mitochondrial state of GCs directly influences the ovarian reserve [68]. Enhanced ROS levels, reduced mitochondrial membrane potential (MMP, an index of mitochondrial function), and decreased mitochondrial DNA (mtDNA) copy number were observed in women with DOR [69]. Because mitochondria are active, their by-products, such as ROS, can intervene to natural cell function by damaging DNA and/or proteins in cells. Thus, increased ROS levels are associated with adverse follicular growth. In humans, mutated mtDNA is strongly associated with ageing phenotypes and reduced lifespan [70]. Introducing functional mtDNA or increasing the amount of mtDNA produced by resveratrol leads to the restoration of ovarian health [71, 72]. A study focusing on mitochondrial biogenesis via cells in the follicle found that mtDNA copy number is downregulated in both GCs and oocytes of DOR patients, and the expression level of PPARGC-1 A (a transcriptional co-activator regulating mitochondrial biogenesis and activity), POLG (encoding the enzyme synthesizing mtDNA), OPA1 (involved in mitochondrial dynamics) and TFAM (a transcription factor related to mtDNA transcription and replication) is decreased significantly in GCs of human with DOR [73]. Therefore, it is obvious that the dysfunction of mitochondrial biogenesis has a detrimental effect on the ovarian reserve and ultimately induces infertility.

### Possible mechanism of incongruous metabolism in DOR

**Fig. 2:**
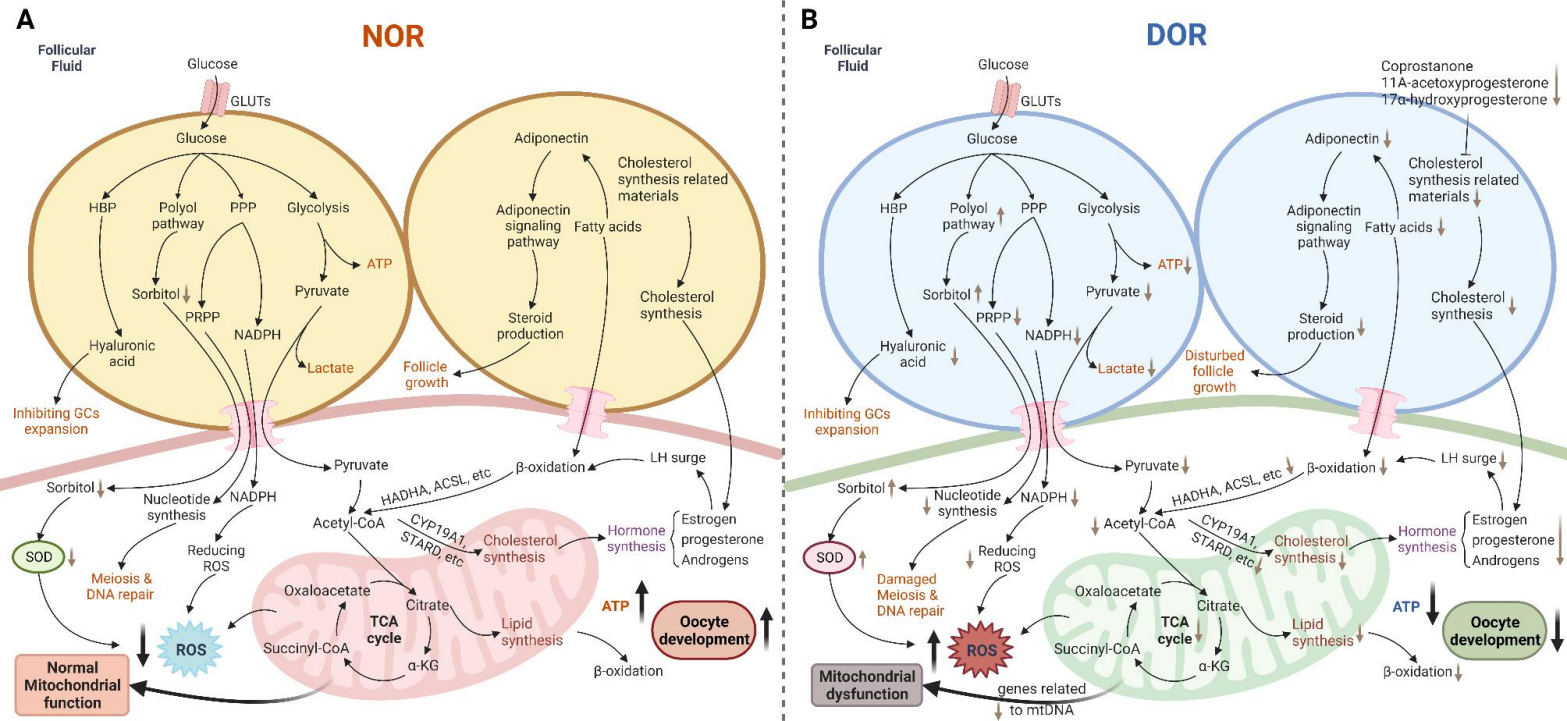
The interconnected loop of glucose, lipids cholesterol and mitochondrial metabolism. **A** Well-function of glucose, lipids, cholesterol and mitochondrial metabolism in maintaining ovarian reserve. **B** Abnormal metabolism of glucose, fatty acids, and cholesterol in the DOR model. Glycolysis, the PPP, and the HBP were suppressed because of decreased GLUTs expression. Deficiencies of pyruvate, NADPH, and hyaluronic acid will lead to a lack of ATP, increased ROS level, damage of meiosis and DNA repair, and inhibition of GCs expansion. The activation of the polyol pathway can enhance sorbitol levels, leading to up-regulated SOD level and impairs mitochondrial function. A lack of fatty acids reduces the function of β-oxidation and the cholesterol synthesis in oocyte, which lead to insufficient of steroid hormones. Decreased estrogen cannot stimulate LH surge-induced β-oxidation. Insufficient of steroid hormones and ATP caused by these metabolic pathways can disturb follicle growth. Mitochondria is the pivot of glucose and lipids metabolism, and its dysfunction can impair these processes

These orchestrated metabolic pathways play an initial role in maintaining the ovarian reserve (Fig. 2A). According to previous studies and the mechanism of metabolism in ovarian function, we hypothesized that the bidirectional communication of glucose, lipids, and cholesterol in the COC may illustrate a possible mechanism of DOR (Fig. 2B): First, during glucose metabolism, the expression of GLUTs in the cytomembrane of GCs may decrease, which may induce a lack of glucose in the GCs. Thus, glycolysis, the PPP, and the HBP were suppressed. This suppression results in deficiencies of pyruvate, NADPH, and hyaluronic acid. However, the polyol pathway is likely to activate by some potential factors which can enhance sorbitol levels. Second, fatty acids have been found in patients with DOR, and a lack of fatty acids reduces the function of β-oxidation, and reduces the cholesterol synthesis in oocyte. Decreased cholesterol synthesis in COC lead to insufficient of steroid hormones, and decreased estrogen cannot stimulate LH surge-induced β-oxidation. Third, mitochondrial dysfunction is an important cause of DOR, as it is the pivot of glucose and lipids metabolism. Furthermore, pyruvate and fatty acids transported to the oocytes are converted to acetyl-CoA, which participates in the TCA cycle to generate sufficient ATP for follicular development. However, this process is abnormal in the DOR. Overall, these dysfunctions are related to a lack of energy, insufficient substances, and rising ROS, and ultimately induce abnormal follicle growth, which is a feature of DOR.

### Maternal influences on primordial follicular pool of the fetus

The embryonic period plays a key role in the establishment of ovarian function. The ovaries of female human newborn babies contain approximately 1–2 million of non-growing follicles, which is a fixed and established number. The count of NFGs or even reserved oocytes later decreases due to follicular atresia and apoptosis with aging, and finally, the decline reaches its culmination in menopause [74–76]. Therefore, the proportion of ovarian reserve achieved before birth determines the later ovarian reserve and limits the reproductive lifespan of a female [77]. This section focusing on the maternal factors that can affect primordial follicular pool of their offspring.

Because the intrauterine environment is closely related to fetal growth and development, an unhealthy intrauterine environment can have a detrimental effect on a newborn’s ovarian reserve. For example, some studies have found that maternal testosterone treatment [78], high gestational weight gain and smoking during pregnancy [79, 80], as well as exposure to aristolochic acid I [81] and D-galactose exposure [82], which are associated with an abnormal intrauterine environment, may interfere with the primordial folliculogenesis of the fetal ovary.

Dietary habits before or during pregnancy can affect the reproductive function of offspring. Studies in animal models have suggested that maternal undernutrition results in a smaller ovary in the fetus and an increased testosterone concentration [83, 84]. In addition, protein restriction in mothers has been shown to impair germ cell and blood vessel development in the fetal ovaries of sheep and the number of primordial follicles in mice [85, 86]. The second study also found that primordial follicles were significantly depleted by 37% at PN21 and 51% at 24-week mice with statistical significance. It has been suggested that the effects of inadequate protein intake may persist for two generations or more, based on evidence that low-protein grandmother diets lead to DOR and a rapid decline in ovarian telomere length [87]. However, if the offspring are exposed to a maternal obesogenic (high-fat or high-sugar) diet, their ovarian reserve will likely be depleted as they grow up [88]. Moreover, maternal caffeine intake during pregnancy is negatively associated with the ovarian reserve in offspring [89].

The ovarian reserve of the offspring is also likely to be affected by the socio-economic environment in which the mothers live [90]. Lower ovarian reserve in child with maternal socio-economic disadvantages may be caused by exposure to unhealthy behaviors, polluted air and poor living conditions in socio-economically disadvantaged areas, resulting in an unsuitable intrauterine growth environment. Evidence from a US farm demonstrated that prenatal exposure to the farm environment (reflecting an uninhabitable living environment) is associated with lower anti-Müllerian hormone concentrations in adulthood, supporting the link between maternal living conditions and the ovarian reserve of their children [91]. Furthermore, exposure to high environmental temperatures impairs the establishment of ovarian reserves in offspring [92]. Chronic inflammation of mother induces intrauterine growth restriction in the fetuses and reduction in the follicles in primordial follicular pool through premature cell apoptosis [93].

**Fig. 3:**
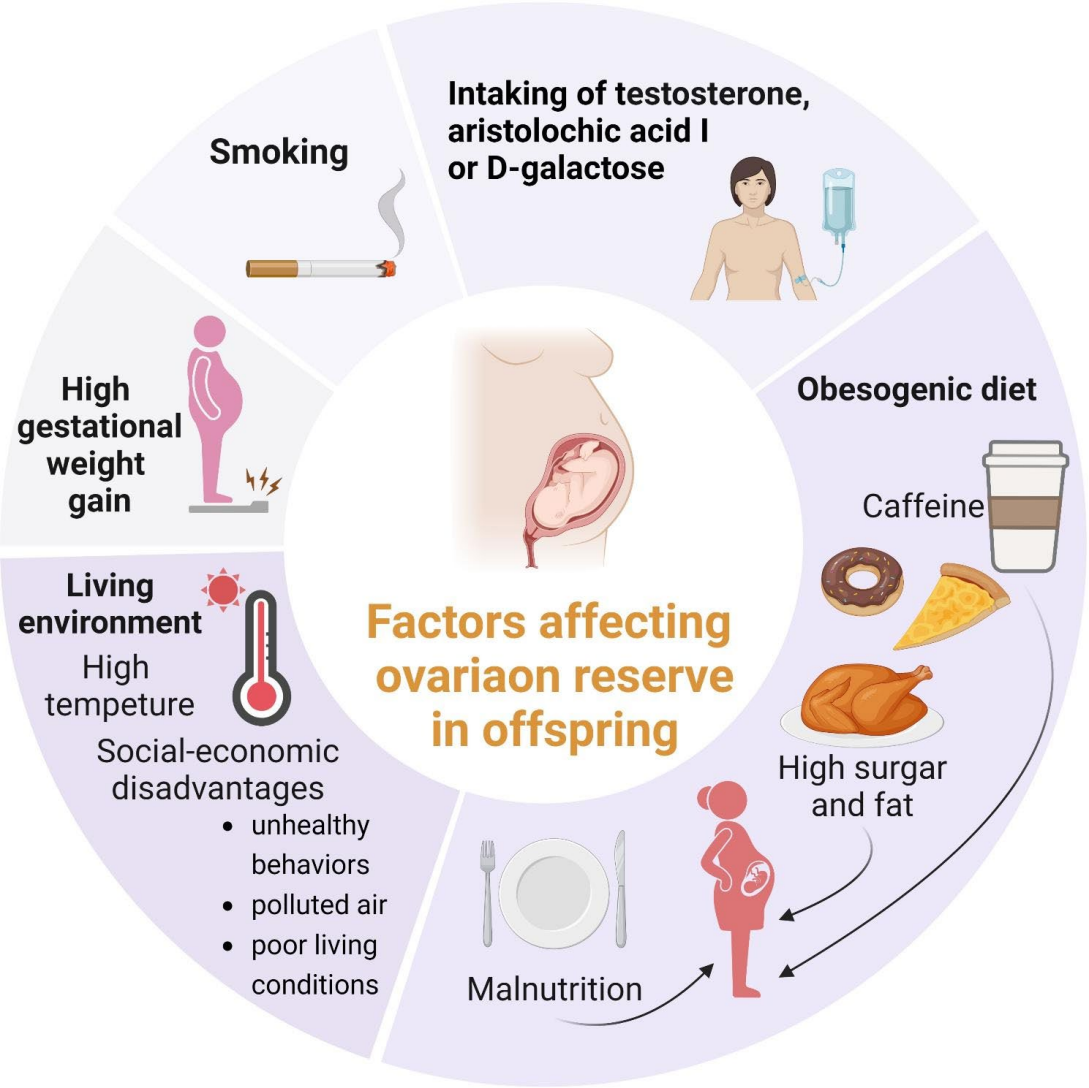
Summary of DOR caused by maternal reasons. Maternal risks that impair ovarian reserve of their offspring have been summarized, including high gestational weight gain, smoking, treatments, diet, and living environment

All in all, these maternal influences on primordial follicular pool of fetus have been summarized in Fig. 3. Since the promising applications and investigations of artificial intelligence in reproductive medicine [94], scientists can contribute a digital model, which depends on the combination these risk factors and clinical symptoms, to predict how much can maternal factors affects their offspring’s ovarian reserve.

### Internal and external factors affecting ovarian reserve

#### Follicle fluid

**Fig. 4:**
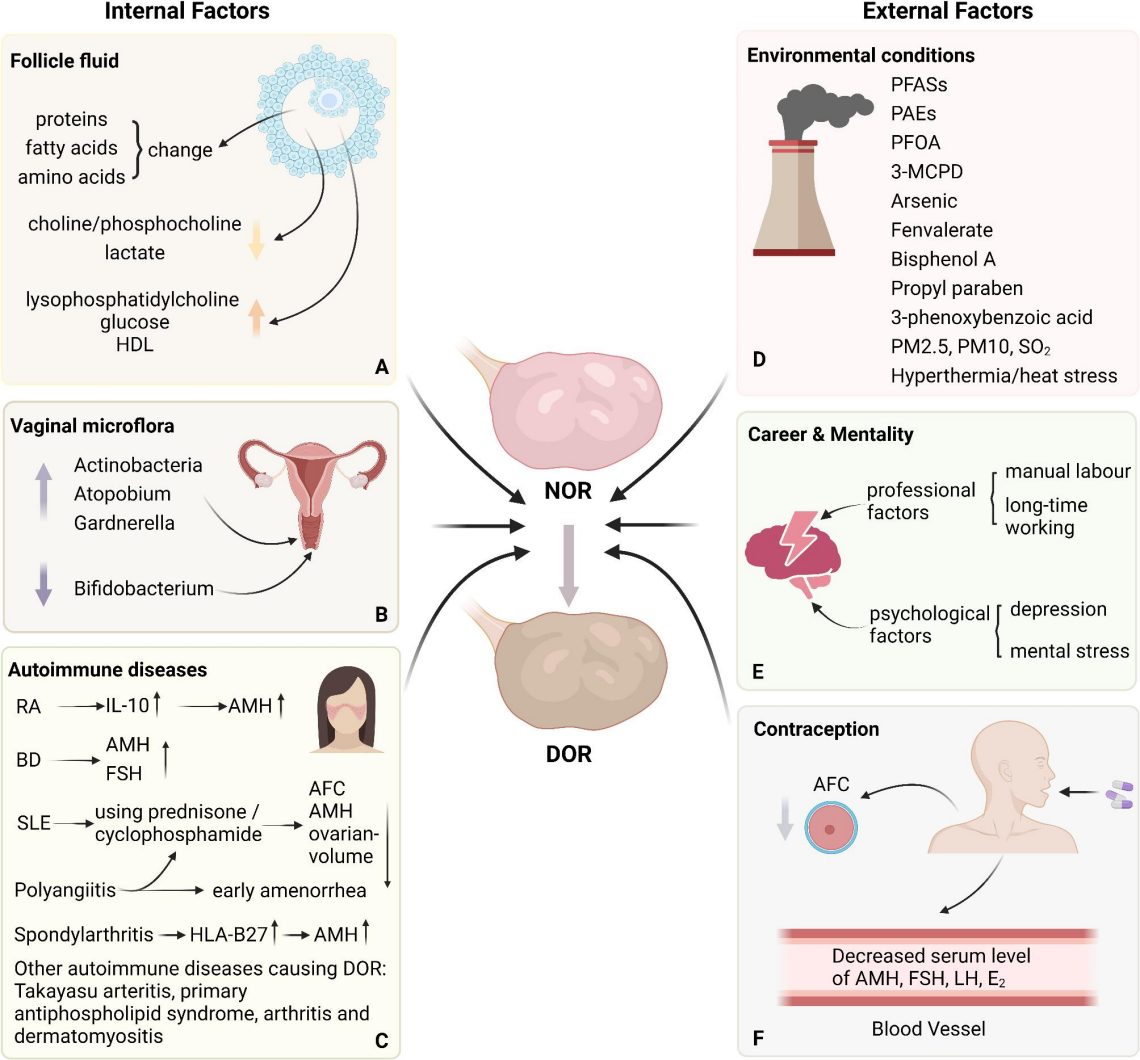
The influence of ovarian reserve caused by internal and external factors. **A** The internal factors including changes of contents in follicle fluid, vaginal microflora alteration, and autoimmune diseases. In follicular fluids, the concentration of proteins, fatty acids, and amino acids, the decreased choline/phosphocholine and lactate, and increased lysophosphatidylcholine, glucose, and HDL are related to DOR. **B** The Actinobacteria, Atopobium and Gardnerella are increased in vagina of patients with DOR while Bifidobacterium is decreased. **C** Some autoimmune diseases can lead to DOR by regulating the level of related hormones. **D** The external factors including environmental pollution, psychological problems, and taking contraception. Living conditions such as persistent organic pollutants, polluted air, and heat stress can affect ovarian reserve by disturbing follicular development, inducing ovarian fibrosis, enhancing GCs apoptosis, and up-regulating inflammatory factors expression. **E** Psychological factors are potential influence of ovarian reserve. **F** Taking contraception can reduce the serum level of AMH, FSH, LH, and E2, and AFC.

The follicle is a unique microenvironment, and as the immediate contact environment of the follicle, follicular fluid plays an essential role in providing the necessary nutrients and material for follicle maturation and oocyte growth. Previous studies have revealed the presence of various proteins associated with reproductive disorders [95, 96]. Refinement of plasma proteins, which make up a significant proportion of the protein in the follicular fluid, showed that the remaining fractions were mainly classified as inflammation-related proteins, complement factors, and proteins involved in lipid metabolism [96, 97]. Fatty acids are important components of the follicular fluid. For example, higher linolenic acid, lower palmitic acid, the concentration of saturated fatty acids, including arachidic acid, erucic acid, tricosanoic acid, and lignoceric acid, and an unbalanced ratio of n-6: n-3 polyunsaturated fatty acids may negatively affect oocyte quality [98, 99]. Follicular T_4_ and P_4_ levels are related to several fatty acids, including arachidonic acid, pentadecanoic acid and heptadecanoic acid [98]. In addition, higher lysophosphatidylcholine levels in follicular fluid are negatively associated with follicle growth [100]. The role of amino acids in the follicular fluid cannot be underestimated. L-alanine, glycine and L-glutamate are positively correlated with oocyte quality, and their deficiency is related to later abnormal blastocyst development [98]. However, in vitro, the high turnover of amino acids, especially valine and isoleucine, in oocytes reflect decreased developmental potential [101]. Moreover, decreasing lactate and choline/phosphocholine concentrations and increasing glucose levels and high-density lipoprotein (HDL) levels in follicular fluid are related to the dysfunction of oocyte development [102]. The concentration of hormones such as AMH and progesterone in the follicular fluid affects oocyte quality [103]. Most importantly, these studies will allow investigation of the relationship between follicular fluid content and ovarian reserve (Fig. 4A).

#### Vaginal microflora

The vaginal microbiota is relative to women of reproductive age, and its variation is significantly correlated with the decline in ovarian reserve [104]. Using AMH, inhibin B, FSH, and LH as ovarian reserve biomarkers, Actinobacteria, Atopobium and Gardnerella were found to be negatively associated with AMH and inhibin B and positively associated with FSH and LH. However, Bifidobacterium exhibited the opposite effect. Its levels were positively associated with AMH levels and negatively associated with FSH and LH levels. This finding agrees with the need to focus on the effects of dysbacteriosis of in vaginal environment on oocyte reserves (Fig. 4B).

#### Autoimmune diseases

Autoimmune diseases are also associated with reduced fertility. The number of AFC, AMH level and determination of ovarian volume were significantly decreased in systemic lupus erythematosus (SLE) patients [105, 106], and the AMH level is related to the duration of the disease and The Systemic Lupus International Collaborating Clinics damage index [107], which is a criterion for the diagnosis and severity of SLE. Another study indicated that disease activity did not seem to affect the ovarian reserve and adrenal gland hormones in adult female SLE patients; the median ovarian volume was significantly lower in SLE patients with current prednisone use [108]. Childhood SLE patients treated with cyclophosphamide had higher median FSH levels and lower median AMH and AFC levels in adulthood than those not treated with cyclophosphamide [109]. Moreover, exposure to and accumulation of cyclophosphamide affect ovarian function and lead to DOR in women with SLE [110] (Fig. 4C). However, as described in an article on disease severity and ovarian reserve tests, it is difficult to determine whether the disease itself or its treatment caused the outcome [111].

A recent systematic review and meta-analysis involving 679 patients with rheumatoid arthritis (RA) and 1,460 controls showed that patients with RA have lower AMH levels [112]. A previous study demonstrated that the AMH level was lower in the RA group, but there was no significance of AMH levels in the activity rheumatoid arthritis, rheumatoid factor (RF), anti-cyclic citrullinated peptide (anti-CCP), erosions, C-reactive protein (CRP) level or therapeutic schedule [113]. Another study reported a similar result, but found a negative correlation between the levels of AMH and IL-10 [114]. Therefore, we suggest that the decrease in AMH levels in RA patients may not be caused by RF but may be related to a more common inflammatory factor caused by RA, and IL-10 is one of the possibilities (Fig. 4C).

Women with Bechet’s disease (BD) have lower serum AMH levels and higher FSH levels [115], indicating that infertility in these patients may be due to decreased ovarian reserve; however, another study focusing on similar research found no difference [116] (Fig. 4C). As a specific biomarker of spondylarthritis, the expression of HLA-B27 is negatively associated with AMH levels, suggesting an adverse impact on the ovarian reserve in the patients [117] (Fig. 4C). A study involving 42 women with polyangiitis who were receiving oral cyclophosphamide therapy found that these women had significantly lower AMH levels, higher FSH levels, and the possibility of early amenorrhea, which conforms to the DOR standard, indicating that exposure to cyclophosphamide is likely to result in decline in the ovarian reserve [118] (Fig. 4C). Additionally, a tendency for the ovarian reserve to decline has been observed in Takayasu arteritis [119], primary antiphospholipid syndrome [120], arthritis [121], and dermatomyositis [122] (Fig. 4C).

In conclusion, it is still controversial whether infertility due to autoimmune diseases is associated with reduced ovarian reserve function; however, the correlation between autoimmune diseases and DOR cannot be ruled out.

#### Environmental exposure

Life events can affect organ function at the ovarian reserve level [123, 124] (Fig. 4D). Because environmental pollution and unhealthy lifestyles are becoming global problems, it is advisable to pay more attention to the effects of environmental pollution, changes in ambient temperature, and poor habits on the reproductive capacity of women.

Persistent organic pollutants caused by pervasive contaminants in drinking water, food, everyday packaging materials, and other contact substances can also reduce ovarian reserve. Exposure to perfluoroalkyl and polyfluoroalkyl substances (PFASs) causes the depletion of follicular cells, promoting menopause and infertility [125]. The influence of PFASs is dose-dependent and leads to decreased serum levels of estradiol and progesterone and apoptosis of oocyte [126, 127]. The toxicity of 3-monochloro-1,2-propanediol (3-MCPD) profoundly affects female ovarian function, including a lower ovary/body ratio, regulation of follicular development, and AFC, higher ovarian fibrosis, GCs apoptosis, and increased expression of inflammatory factors [128]. Moreover, corticosterone and cortisol in FF of women expose to phthalates (PAEs), a plasticizer which can release into environment, are decreased significantly [129].

Individuals may unconsciously be exposed to toxic substances. For instance, a high concentration of arsenic has been found in women with DOR, and arsenic inhibits the expression of steroidogenic factor-1 by upregulating the DNA methylation level of its promoter region resulting in decreased expression of relative proteins, including STAR, CYP11A1 and CYP19A1, which are important genes related to lipid metabolism during follicle growth [130]. Chronic exposure to propyl paraben [131], 3-phenoxybenzoic acid [132], or bisphenol A [133] was strongly associated with abnormal laboratory test results, indicating potential toxicity to the ovarian reserve. Fenvalerate, an insecticide widely used in modern agriculture, inhibits follicle expansion by interfering with steroidogenesis by downregulating STAR and P450 side chain cleavage enzyme expression [134]. A recent study has also found that perfluorooctanoic acid (PFOA) is enriched in the FF of patients with DOR and that the metabolic composition of FF is affected by PFOA [135].

Increasing attention is being paid to the effects of the living environment on ovarian reserve. There is an inverse relationship between outdoor air pollution (PM2.5, PM10 and SO_2_) and the reduction in AFC or AMH [136–139]. Previous studies have illustrated that the development of ovarian follicles and oocyte competence are counteracted by hyperthermia or heat stress [140, 141]. In humans, with an average increase in the ambient temperature of around 1 °C, AFC decreases by 1.6%, indicating that heat is a negative factor in ovarian reserve [142]. As environmental stress, heat stress induces the apoptosis of ovarian cells through several processes, such as enhancing the expression level of BCL2L1(regulator of apoptosis during mammalian ovarian maturation) [141] and miR-33 (factor suppressing VEGF signaling) [143], regulating heat stress proteins (HSP70 and HAPA13) levels [144], and activating the FasL/Fas and TNF-a systems [145]. Heat stress also alters glucose, cholesterol and non-esterified fatty acid levels in FF [146], greatly reducing gonadotropin receptor expression in GCs [147].

#### Psychological factors

Studies focusing on occupational factors have shown that women working hard and long hours, especially those who have to handle heavy objects and work non-daily shifts, have lower ovarian reserve [148]. Evidence shows that this negative effect may promote a decline in AFC in women [149]. Furthermore, women with a history of depression have significantly higher FSH levels, and psychological stress is negatively related to AMH levels in females with statistical significance [150]. On the other hand, DOR women with higher self-esteem may have less fertility distress and a better effect on reproductive outcome [151]. Therefore, we hypothesized that the working model, as one of the most common daily influences, may be closely related to the mental state of professional women, and poor psychological conditions, such as fatigue, anxiety, despondency or tension, are a vulnerability factor in DOR, or optimism is a protective factor in maintaining the ovarian reserve. Part E of Fig. 4 have shown above results.

#### Contraception

With the development of pharmaceutical science and female awareness, contraception usage to avoid pregnancy is becoming more common among women aged 15–50 [152]. It is reported that almost 90% of sexually active women who do not want to become pregnant use contraception [153], demonstrating that contraception is an influential element of DOR. An investigation involving 887 women using or not using oral contraception found a significant decrease in AMH levels and a reduced AFC of approximately 5-7 mm and 8-10 mm in diameter, after adjusting for age, BMI, smoking and maternal age at menopause [154]. A study with a large number of recruits suggested a similar result: women who use hormonal contraceptives or combined oral contraceptive pills have a lower mean AMH level than women who do not use contraceptives [155]. Moreover, hormonal contraception can impair AFC, ovarian volume, and ovarian vascular indices and reduce serum FSH, LH, and E_2_ levels [156, 157] (Fig. 4F).

### Possible intervention in the future

Assisted reproduction technique (ART) is a final method for infertilities, whereas, studies have found that these treatments may lead to adverse outcomes for their babies, who are more likely to have neuro-psycho-motor malfunction, high risk of small for gestational age babies, low birthweight, preterm birth (PTB), and congenital heart diseases [158–160]. Previous studies have summarized that cell or gene therapies can resuscitate ovarian function which is affected by internal and external factors [161, 162]. Thus, it is beneficial to find some new methods to intervene in patients themselves rather than blindly rely on ART, and this part put forward possible pointcuts.

## Conclusion

This review provides clear and convicting evidence that the mechanism of the DOR is so intricate and ambiguous that it must be rearranged and recognized. These results would be conducive to a better understanding of the mechanisms and influence of follicular development and ovarian reserves. Collectively, subsequent studies may look at aspects of mechanistic studies on how sex hormones and metabolism affect female ovarian reserve, disease, risk factors, and maternal levels of certain hormones during pregnancy; advanced studies on the elimination of risk factors in living conditions; and social studies on how to pay more attention to women’s mental health.

## Data Availability

The datasets generated and/or analyzed during the current study are available in the MEDLINE repository. https://pubmed.ncbi.nlm.nih.gov/. All figures were depicted by BioRender. https://www.biorender.com/.

## Abbreviations

DOR: Diminished ovarian reserve
NOR: Normal ovarian reserve
GCs: Granulosa cells
AFC: Antral follicle count
AMH: Antimüllerian hormone
FSH: Follicle-stimulating hormone
LH: Luteinizing hormone
E2: Estrogen
P: Progesterone
AMHR: Antimüllerian hormone receptor
AR: Androgen receptor
FSHR: Follicle-stimulating hormone receptor
LHR: Luteinizing hormone receptor
ER: Estrogen receptor
PR: Progesterone receptor
FF: Follicular fluid
GLUT: Glucose transporter
COC: Cumulus-oocyte complexes
PPP: Pentose phosphate pathway
HBP: Hexosamine biosynthetic pathway
ROS: Reactive oxygen species
PRPP: Phosphoribosyl pyrophosphate
SOD: Superoxide dismutase
cAMP: Cyclic adenosine monophosphate
MMP: Mitochondrial membrane potential
mtDNA: Mitochondrial DNA
HDL: High-density lipoprotein
SLE: Systemic lupus erythematosus
RA: Rheumatoid arthritis
RF: Rheumatoid factor
anti-CCP: Anti-cyclic citrullinated peptide
CRP: C-reactive protein
BD: Bechet’s disease
PFASs: Polyfluoroalkyl substances
PFOA: Perfluorooctanoic acid
ART: Assisted reproduction technique
